# Effect of divergence in residual methane emissions on feed intake and efficiency, growth and carcass performance, and indices of rumen fermentation and methane emissions in finishing beef cattle

**DOI:** 10.1093/jas/skab275

**Published:** 2021-10-01

**Authors:** Paul E Smith, Sinead M Waters, David A Kenny, Stuart F Kirwan, Stephen Conroy, Alan K Kelly

**Affiliations:** 1 Teagasc, Animal and Bioscience Research Department, Animal and Grassland Research and Innovation Centre, Grange, Dunsany, County Meath, Ireland; 2 UCD School of Agriculture and Food Science, University College Dublin, Belfield, Dublin 4, Ireland; 3 Irish Cattle Breeding Federation, G€N€ IR€LAND Progeny Test Centre, Tully, Kildare Town, County Kildare, Ireland

**Keywords:** beef cattle, residual methane emissions

## Abstract

Residual expressions of enteric emissions favor a more equitable identification of an animal’s methanogenic potential compared with traditional measures of enteric emissions. The objective of this study was to investigate the effect of divergently ranking beef cattle for residual methane emissions (**RME**) on animal productivity, enteric emissions, and rumen fermentation. Dry matter intake (**DMI**), growth, feed efficiency, carcass output, and enteric emissions (GreenFeed emissions monitoring system) were recorded on 294 crossbred beef cattle (steers = 135 and heifers = 159; mean age 441 d (SD = 49); initial body weight (BW) of 476 kg (SD = 67)) at the Irish national beef cattle performance test center. Animals were offered a total mixed ration (77% concentrate and 23% forage; 12.6 MJ ME/kg of DM and 12% CP) ad libitum with emissions estimated for 21 d over a mean feed intake measurement period of 91 d. Animals had a mean daily methane emissions (**DME**) of 229.18 g/d (SD = 45.96), methane yield (**MY**) of 22.07 g/kg of DMI (SD = 4.06), methane intensity (**MI**) 0.70 g/kg of carcass weight (SD = 0.15), and RME 0.00 g/d (SD = 0.34). RME was computed as the residuals from a multiple regression model regressing DME on DMI and BW (*R*^2^ = 0.45). Animals were ranked into three groups namely high RME (>0.5 SD above the mean), medium RME (±0.5 SD above/below the mean), and low RME (>0.5 SD below the mean). Low RME animals produced 17.6% and 30.4% less (*P* < 0.05) DME compared with medium and high RME animals, respectively. A ~30% reduction in MY and MI was detected in low versus high RME animals. Positive correlations were apparent among all methane traits with RME most highly associated with (*r* = 0.86) DME. MY and MI were correlated (*P* < 0.05) with DMI, growth, feed efficiency, and carcass output. High RME had lower (*P* < 0.05) ruminal propionate compared with low RME animals and increased (*P* < 0.05) butyrate compared with medium and low RME animals. Propionate was negatively associated (*P* < 0.05) with all methane traits. Greater acetate:propionate ratio was associated with higher RME (*r* = 0.18; *P* < 0.05). Under the ad libitum feeding regime deployed here, RME was the best predictor of DME and only methane trait independent of animal productivity. Ranking animals on RME presents the opportunity to exploit interanimal variation in enteric emissions as well as providing a more equitable index of the methanogenic potential of an animal on which to investigate the underlying biological regulatory mechanisms.

## Introduction

Global food production has benefited from the ability of ruminant livestock to convert plant matter into high-quality sources of dairy and meat protein for human consumption (Waters et al., 2020). However, ruminant, relative to monogastric, derived food products have a much greater carbon intensity (Herrero and Thornthon, 2013), with methane originating from domesticated cattle accountable for ~4.5% of anthropogenic emissions (Gerber et al., 2013). Consequently, mitigation strategies to reduce enteric methane emissions from cattle have been a key research priority for livestock scientists in recent decades. Numerous dietary interventions (strategic supplementation with various feedstuffs and bioactive compounds, combined with animal management approaches) have been advocated to offer potential methane mitigation solutions to livestock producers (Hristov et al., 2013; Beauchemin et al., 2020; Honan et al., 2021); however, a supplement with consistent antimethanogenic properties, and no adverse implications to animal performance, is yet to be made commercially available.

Enteric methane emissions is a trait which is moderately heritable (*h*^2^ = 0.23 to 0.30) (Pinares-Patiño et al., 2013; Donoghue et al., 2016; Manzanilla-Pech et al., 2016) with large interanimal inherent variation, presenting the possibility of cumulative and permeant reductions in ruminant livestock derived emissions through genetic selection as an alternative mitigation solution (Wall et al., 2010; Pickering et al., 2015; de Hass et al., 2017; Beauchemin et al., 2020). Nonetheless, determining the optimal low methane phenotype, with which to select cattle, poses a challenge due to the relationship of methanogenesis with other traits of importance to animal productivity (de Hass et al., 2017). Feed intake and daily methane emissions (**DME**; g/d) are both phenotypically (Herd et al., 2014) and genetically (Donoghue et al., 2016; Manzanilla-Pech et al., 2016) associated. As a result, the implementation of breeding strategies, where DME is the targeted phenotype, will likely result in a concurrent reduction to voluntary feed intake, and subsequently animal performance, in future generations of livestock (Herd et al., 2014; de Hass et al., 2017).

Selection on the basis of methane emissions expressed as a proportion of feed intake (methane yield; **MY**) has been the traditional selection approach, as the trait was previously perceived to be free from any association with feed intake or body weight (**BW**) but positively correlated with DME, when open-circuit respiration chambers and restricted feed intake were utilized as reference methodology for quantifying enteric emissions (Herd et al., 2014; Donoghue et al., 2016). However, the selection of animals on the basis of ratio traits has been disputed by virtue of their unpredictable response to other traits of economic importance in beef production (Pickering et al., 2015). In addition, a negative phenotypic correlation between MY and feed intake has recently been observed across both concentrate and forage based diets under ad libitum feeding conditions with the use of the GreenFeed emissions monitoring system (Bird-Gardiner et al., 2017; Renand et al., 2019).

Consequently, due to the aforementioned shortcomings, there has been increasing interest in the use of the residual methane emissions (**RME**) concept to identify animals with a greater genetic propensity for lower methane output, principally due to its ability to overcome the limitations associated with proportional expression of methane emissions relative to other traits and by design, its lack of relationship with feed intake. RME can be defined as the difference in the animals actual and expected methane output, based on its level of feed intake and BW (Bird-Gardiner et al., 2017). First proposed by Herd et al. (2014), the trait has been observed to be phenotypically and genetically independent of feed intake and bodyweight (Herd et al., 2014; Donoghue et al., 2016; Bird-Gardiner et al., 2017). Indeed, the independence of RME from animal productivity, also affords the opportunity to unravel the inherent variation in underlying biological mechanisms influencing methanogenesis. Currently, there is a paucity of information on the implications of ranking commercially representative beef cattle for RME on animal productivity, feed efficiency, and carcass output.

Therefore, the objectives of this study were to 1) investigate the effects of divergently ranking beef cattle for RME on DME, yield, intensity, animal productivity, and rumen fermentation; 2) examine the phenotypic relationships of RME with other traits of economic importance to beef production.

## Materials and Methods

All animal procedures used in this study were approved by the Teagasc Animal Ethics Committee and conducted using procedures consistent with the experimental license (AE19132/P078) issued by the Irish Health Products Regulatory Authority in accordance with European Union legislation (Directive 2010/63/EU), for the protection of animals used for scientific purposes.

### Animal management and performance test

Over a period of 18 mo, data were obtained from 294 commercial beef cattle (steers = 135 and heifers = 159; mean age 441 d (SD = 49)) enrolled in a feed efficiency performance test. Cattle were the progeny of AI bulls, under evaluation as part of the Gene Ireland Breeding Program (https://www.icbf.com/?page_id=12900), and were recruited from commercial breeding herds, based on factors including sire, breed, genetic merit, pedigree, and age, and performance tested under standardized conditions at the Irish Cattle Breeding Federation (ICBF) national beef bull progeny test station (Tully, Co. Kildare). Cattle included in this study originated from continental late maturing (**LM**) beef dams (Charolais, Limousin, or Simmental), sired by early maturing (**EM**) or LM sire breeds. The proportion of EM and LM sired animals was 25% and 75%, respectively.

Eligible cattle enter the test center in groups of 40 to 75 cattle, hereby referred to as “batches”, and undergo a minimum 100 d feed efficiency performance test. Starting in January 2019 and finishing in July 2020, animals from seven consecutive batches were included in this study. Upon arrival at the facility, cattle were allocated to indoor pens (6.1 m × 4.6 m) bedded with peat. Cattle were separated based on gender and initially penned in groups of five to six depending on their initial weight and age. Cattle were offered a 30 d adjustment period to allow dietary acclimatization and adaption to the facilities. Within the first week of arrival at the test center, animals were fitted with a radio frequency identification tag (HDX EID Tag, Allflex Livestock Intelligence, Dallas, TX). Once tagged, pen size was increased by opening the gates between adjacent pens to accommodate 11 to 30 animals per pen with animals comingled for a minimum 21 d period, prior to the beginning of the feed intake measurement period. This was done to facilitate the measurement of enteric methane production (discussed later). After the adjustment period, animals were subjected to a mean daily feed intake measurement period of 91 d (71 to 128 d). The mean age and BW of animals at the beginning of the test was 441 d (SD = 49) and 476 kg (SD = 67), respectively. Steers and heifers averaged 476 (SD = 46) and 410 (SD = 27) days of age while LM and EM averaged 442 (SD = 51) and 435 (SD = 43) days of age at the commencement of the measurement period, respectively. Post completion of their performance test, cattle were slaughtered in a commercial abattoir.

### Measurement of feed intake and chemical composition

Individual daily feed intake was recorded with the use of electronic feeding stations (RIC Feed-Weigh Trough; Hokofarm Group BV, Marknesse, The Netherlands) with a feeding event recorded with each 100 g fluctuation in weight at the feed bunk. The mean duration of the feed intake measurement was 91 d and ranged from 71 to 128 d. Cattle were offered ad libitum access to the same total mixed ration (**TMR**) diet (77% concentrate and 23% grass hay). The TMR consisted of 3 kg of hay and 10 kg of concentrates, mixed with 9 kg of water. The ingredient composition of the concentrate was as follows (DM basis); maize meal 28%, barley 24%, soya hulls 14%, dried distillers grains 10%, maize gluten meal 9%, soya bean meal 5.5%, molasses 5%, mineral and vitamin premix 3.75%, vegetable oil 0.7%, and yeast 0.05%. The concentrate was a pelleted ration, formulated to have a crude protein (**CP**) content of 140 g kg and had a predicted ME content of 12.6 MJ/kg DM (NRC, 2016). A fresh TMR was prepared daily which was both mixed and administered via a feed wagon. Feed was offered once per day and at all times animals had unrestricted access to clean drinking water.

Samples of both the TMR diet and concentrates were obtained weekly and stored at −20 °C for laboratory analysis. Feed samples were defrosted overnight in a refrigeration unit (4 °C) prior to analysis. The dry matter (**DM**) of TMR and concentrate samples was determined after drying at 90 °C for 16 h in a forced-air circulation oven. For chemical analysis, TMR and concentrate samples were oven dried at 40 °C for 48 h and then ground through a 1 mm screen (Willey mill; Arthur H. Thomas, Philadelphia, PA). After grinding, samples collected during each intake run were pooled, respectively.

Ash concentrations (g/kg DM) were determined by complete combustion in a muffle furnace (Nabertherm, GmbH, Lilienthal, Germany) at 550 °C for 4 h. Nitrogen concentration (g/kg DM) of the feed was determined using a LECO 828 Series Macro Combustion instrument (Leco Instruments, UK, Ltd, Stockport, UK). The nitrogen concentration of the feed was multiplied by 6.25, to determine CP concentrations (g/kg DM). Neutral detergent fiber (**NDF**) and acid detergent fiber (**ADF**) concentrations were determined by the method of Van Soest et al. (1991) using the ANKOM220 Fiber Analyzer (ANKOM Technology, Macedon, NY). TMR and concentrate samples were analyzed for NDF with sodium sulfite and with a heat stable amylase included for both sets of samples. NDF and ADF are expressed inclusive of residual ash (g/kg DM). Gross energy was determined on pelletized samples using a bomb calorimeter (Parr Instrument Company, Moline, IL). Ether extract was determined using Soxtec instruments (Tecator, Höganäs, Sweden) and light petroleum ether. The chemical composition of the TMR and concentrate ration are displayed in Table 1.

**Table 1.** Details of the chemical composition of total mixed ration (TMR) and concentrates offered during feed efficiency and enteric emissions measurement periods (±SD)

	Concentrate	TMR
Chemical composition (% of DM unless stated)		
Dry matter	91.7 (0.8)	50.1 (0.9)
Crude protein	13.8 (0.4)	12.2 (0.3)
Neutral detergent fiber	21.8 (0.7)	33.5 (1.1)
Acid detergent fiber	10.8 (0.3)	17.9 (0.6)
Either extract	3.4 (0.6)	2.3 (0.3)
Ash	7.4 (0.2)	7.3 (0.1)
Gross energy, MJ/kg DM	16.8 (0.3)	16.7 (0.2)

### Animal growth and ultrasonic muscle and fat deposition

At the beginning of each test period, and every 21 to 28 d thereafter until the end of the measurement period, cattle were weighed with a calibrated scales (ID 3000 scales, Tru Test, Ireland). BW measurements were used to derive measures of feed efficiency and daily weight gain for each animal. Pre-slaughter ultrasound measurements of muscle and fat deposition and proportion of intramuscular fat were collected as described by Kelly et al. (2019). Measurements were taken with the use of the same Esaote-Pie Medical Aquila PRO Vet ultrasound scanner, with a 3.5 MHz transducer head, by a trained technician.

### Carcass characteristics

Animals were slaughtered on average 3 d after the completion of the feed efficiency test period in a European Union licensed commercial facility 77 km away (Slaney Foods International, Bunclody, Co. Wexford, Ireland). Animals were slaughtered within 1 h of arrival at the facility. Carcass weight (**CW**) was measured, on average, 2 h post-slaughter. After slaughter, carcass conformation and fat percentage were automatically recorded on a 15 point scale using video imaging analysis equipment (VBS2000; e+v Technology GmbH & Co.KG, Oranienburg, Germany) as described by Hickey et al. (2007).

### Enteric methane and carbon dioxide output

Enteric methane and carbon dioxide measurements were obtained on all animals using the GreenFeed emissions monitoring system (GEM; C-Lock Inc., Rapid City, SD) over 21 consecutive days throughout the feed intake measurement period. The commencement of the emissions estimation period ranged from days 0 to 36 of the feed intake measurement period.

A detailed description of the workings of the GEM has been previously described (Huhtanen et al., 2015; Hammond et al., 2016; Patra et al., 2016; Hristov et al., 2018). Briefly, the concentration of enteric gaseous emissions emitted by individual animals per visit was determined by the GEM software, as a gas flux, from the increase in the concentration of each gas, relative to background levels, accompanied by adjustments for airflow rate and principles of the ideal gas law, and reported in grams per day. The ratio of animals to a single GEM, ranged from 11 to 30 depending on numbers in each intake group.

Each GEM was connected to both a span (0.05% methane (CH_4_), 0.5% carbon dioxide (CO_2_) balanced with zero grade nitrogen gas; BOC Gas, Dublin, Ireland) and zero gas canister (zero grade nitrogen gas; BOC Gas, Dublin, Ireland) with auto calibrations performed every 3 d. Throughout the duration of the experiment, monthly CO_2_ recovery tests were performed, as per the manufactures instructions, to assess the airflow of the unit. A clean air filter was replaced in each unit on a weekly basis or if airflow dropped below 27 L/s. The bait feed utilized to entice animals to use the GEM, was the same pelleted concentrate included in the TMR. Feed drops were weighed on a weekly basis for each GEM unit using the average of 10 feed drops. Throughout the experimental period and across GEM units, CO_2_ recoveries and the weight of individual feed drops averaged 99.32 ± 3.29% and 34.02 ± 4.11 g, respectively. The mean airflow for all data points utilized in this experiment was 37.1 ± 2.59 L/s.

Previously, Arthur et al. (2017) determined a minimum of 30 visits to GEM, of >3 min in length, to be sufficient to accurately determine enteric methane emissions for individual animals. In line with these recommendations, the GEM was programmed to drop 30 g of bait feed, every 35 s, to a maximum of six drops per visit for each animal. Once an animal reached the maximum number of bait feed drops, a minimum 4 h interval was required before an animal could receive another drop of bait feed from the unit.

### Rumen fermentation

During the last week of the enteric emissions measurement period, samples of rumen digesta were obtained from each animal, before feeding, using a transoesophageal rumen sampling device (FLORA rumen scoop; Guelph, Ontario, Canada). Feed was restricted from animals for a minimum of 2 h prior to sampling. After collection, ruminal fluid pH was measured immediately using a digital pH meter (Orion SA 720; Thermo Fisher Scientific, Waltham, MA) followed by the preservation of samples via snap freezing in liquid nitrogen. On the same day of sampling, samples were transported 61 km away to the Teagasc research facility (Teagasc Grange, Dunsany, Co. Meath, Ireland) on dry ice and stored at −80 °C until analysis was conducted.

Rumen fluid samples were thawed on a laboratory bench top and diluted in 50% TCA acid at a ratio of 4:5 in favor of rumen fluid. Following the addition of acid, samples were centrifuged for 10 min (2,000 rpm; 4 °C) after which, 250 μL of supernatant was drawn off into a test tube and diluted with 3.75 mL of dH_2_O and 1 mL of internal standard (0.5 g 3-metyl-*n*-valeric acid in 1 liter of 0.15 M oxalic acid). Following centrifuging for 5 min (2000 rpm; 21 °C), the dilution was filtered through a 0.45 μm filter (Cronus Syringe filter PTFE 13mm; SMI-LabHut Ltd., Maisemore, Gloucester, UK) into a 2 ml GC vial (Thermo Scientific, Langerwehe, Germany) and frozen at −20 °C until VFA analysis.

One microliter of sample was injected by auto sampler on a Varian (Saturn 2000) gas chromatograph (GC) 450 (Varian, Middelburg, The Netherlands) with a 30 m × 0.25 mm i.d. BP21 FFAP capillary column (Trajan Scientific, Milton Keynes, UK). The initial injector temperature was 60 °C for 10 s, rising to 110 °C at a rate of 30 °C/min, this temperature then increased at rate of 10 °C/min to 200 °C (held for 2 min). Helium was used as a carrier gas. The pressure of the column was held at 19.3 pounds per square inch (psi) and the column rate was 17.2 mL/min.

Total short chain fatty acids (**SCFA**) are reported as the sum total of all VFAs (mM). The percentage of acetate, propionate, and butyrate are reported as the proportion of each individual VFA relative to the total SCFA. The ratio of acetate to propionate (**A:P**) was calculated. Estimates of theoretical hydrogen (H) production by each animal at the time of sampling were calculated based on the concentration of individual VFAs as described by Marty and Demeyer (1973) with the exclusion of hydrogen gas (H_2_).

### Traits investigated and their derivations

Average dry matter intake (**DMI**; kg) was calculated as the average daily feed intake of each animal (including the GEM bait feed during the estimation of enteric emissions) over the course of the experiment after correcting for DM as described above. Average daily gain (**ADG**; kg) during the test period for each animal was computed as the coefficient of the linear regression of BW (kg) on time. The weight of the animal at the beginning and end of the feed intake measurement period was used to calculate initial and final live BW, respectively. Mean metabolic BW (**MetBW**; kg) was represented as average test BW^0.75^. Pre-slaughter muscular depth (**MD**; mm), pre-slaughter fat depth (**FD**; mm), and pre-slaughter intramuscular fat (**IMF**; %) were determined using data obtained during ultrasound measurements as previously described. Carcass conformation grade and fat class score values were scaled, with 1 representing the poorest conformation and 15 the best conformation in carcass conformation grade and 1 representing the leanest value and 15 the fattest in fat class scores, respectively (Hickey et al., 2007). Gain to feed ratio (**G:F**) was obtained for each animal by dividing ADG by average DMI.

Residual feed intake (**RFI**) was computed for each animal and was assumed to represent the residuals from a multiple regression model regressing DMI on ADG and MetBW. Each batch of animals was subsequently treated as a contemporary group (**CG**) and included as a fixed effect in the model. The base model used was


$$\begin{array}{l} Y_{j}=\beta_{0}+\beta_{1}MetBW_{j}+\beta_{2}ADG_{j}+CG_{i}+e_{j}, \end{array}$$

where Y_*j*_ is the DMI of the *j*th animal, β _0_ is the regression intercept, β _1_ is the regression coefficient on MetBW, β _2_ is the regression coefficient on ADG, CG_*i*_ is the fixed effect of the *i*th batch of animals, and e_*j*_ is the uncontrolled error of the *j*th animal. The multiple regression model fitted for RFI explained 72% of the variation in DMI while RFI averaged 0.00 kg DM/d (SD = 0.77). RFI values ranged from −3.53 to 2.25 kg/d and represented a difference of 5.78 kg/d between the lowest and highest ranked animals for RFI.

Methane DMI (**MDMI**; kg) was calculated as the sum total of the combined TMR and concentrate supplementation from the GEM for each animal averaged over the methane measurement period. Average daily methane (**DME**; CH_4_ g/d) and carbon dioxide emissions (**CME**; CO_2_ g/d) for each animal was derived from the sum of emissions of each gas per spot measurement divided by the total number of these measurements as recorded by the GEM over the test period. Only spot measurements where the visitation to the GEM was ≥3 min were included in the analysis. MY (CH_4_ g/DMI kg) was calculated for each animal by dividing DME by the MDMI. The weight of individual animals on the 30th day of the feed intake measurement period was used to standardize BW for methane analysis (hereby referred to as methane BW). Individual animal BW on day 30 was calculated based on the regression analysis conducted during the calculation of ADG. Methane per unit of BW (CH_4_ g/BW) and methane intensity (**MI**; CH_4_ g/carcass out kg) were calculated by dividing DME by methane BW and CW (kg), respectively. DME was also expressed per unit of ADG, using ADG calculated over the feed intake test period (**MADG**; CH_4_ g/ADG kg).

Residual methane emissions (**RME**; CH_4_ g/day) was computed for each animal using the equation described by Bird-Gardiner et al. (2017). RME was assumed to represent the residuals from a multiple regression model regressing DME on MDMI and methane BW with CG included as a fixed effect in the model. The base model used was


$$\begin{array}{l} Y_{j}=\beta_{0}+\beta_{1}MDMI_{j}+\beta_{2}methane\;BW_{j}+CG_{i}+e_{j}, \end{array}$$

where Y_*j*_ is the DME of the *j*th animal, β _0_ is the regression intercept, β _1_ is the regression coefficient on MDMI, β _2_ is the regression coefficient on methane BW, CG_*i*_ is the fixed effect of the *i*th batch of animals, and e_*j*_ is the uncontrolled error of the *j*th animal. The multiple regression for RME explained 45% of the variation in DME while RME averaged 0.00 g/d (SD = 34.05). RME values ranged from −114.07 to 84.99 and represented a difference of 199.06 g/d between the lowest and highest ranked animals for RME. SDs above and below the mean were used to group animals into high RME (RME > 0.5 SD above the mean), medium RME (RME ± 0.5 SD above and below the mean), and low RME (RME > 0.5 SD below the mean).

In addition, for comparative purposes among methane phenotypes, RME was calculated using the equation proposed by Renand et al. (2019) whereby DCE replaced DMI. RME with DCE (**RME**_**CO2**_; CH_4_ g/ day) was assumed to represent the residuals from a multiple regression model regressing DME on DCE and methane BW with CG included as a fixed effect in the model. The base model used was


$$\begin{array}{l} Y_{j}=\beta_{0}+\beta_{1}DCE_{j}+\beta_{2}methane\;BW_{j}+CG_{i}+e_{j}, \end{array}$$

where Y_*j*_ is the DME of the *j*th animal, β _0_ is the regression intercept, β _1_ is the regression coefficient on DCE, β _2_ is the regression coefficient on methane BW, CG_*i*_ is the fixed effect of the *i*th batch of animals, and e_*j*_ is the uncontrolled error of the *j*th animal. The multiple regression for RME_CO2_ explained 57% of the variation in DME while RME averaged 0.00 g/d (SD = 30.72). RME values ranged from −96.76 to 94.76 and represented a difference of 191.52 g/d between the lowest and highest ranked animals for RME_CO2_.

### Data and statistical analyses

Raw emissions data were processed by C-Lock Inc. and checked for irregularities. Data were downloaded from the C-Lock Inc. website with an additional round of checks performed to identify and remove outliers as per the methods described by Coppa et al. (2021). To detect outliers, the SD was calculated for both CH_4_ and CO_2_ using all spot measurements (≥3 min) supplied by C-Lock Inc. Following this, spot measurements of CH_4_ were regressed on CO_2_ and vice versa, allowing for the prediction of both gases using the equations generated using the REG procedure in SAS (SAS Inst. Inc., Cary, NC; version 9.4). Residuals were then calculated from the differences of the predicted and observed spot measurements for each gas. Finally, outliers were detected and discounted in the analysis if the residual/SD was >3 for a measurement of either gas. After the removal of outliers, 99.68% of the emissions data were maintained and used for analysis.

Data were checked for normality and homogeneity of variance by histograms, qqplots, and formal statistical tests as part of the UNIVARIATE procedure of SAS. Data from 12 animals were not included in the analysis as visitation to the GEM was below the threshold of 30 visits (*n* = 3) or the data from animals were identified as statistical outliers (*n* = 9). This resulted in a final dataset of 282 animals. A mixed model ANOVA (GLIMMIX procedure of SAS) was used to examine the effect of RME group on performance, intake, feed efficiency, body composition, methane emissions, and ruminal fermentation profiles. The statistical model used included the fixed effect of RME group (high, medium, and low) breed maturity/genotype (LM and EM), sex (steer and heifer), and their interactions. Non-statistically significant (*P* > 0.10) interactions were subsequently excluded from the final model. Age and initial bodyweight at the start of each performance test were included as covariates with each batch of animals treated as a CG and incorporated as a random effect in the statistical model. Differences among means were determined by *F*-tests using type III sums of squares. The PDIFF option and the Tukey test were applied to evaluate pairwise comparisons between means. Mean values were considered to be different when *P* < 0.05 and considered a tendency when *P* ≥ 0.05 and < 0.10. The associations among the traits were determined through partial correlations, adjusted for gender, breed maturity, and CG using the MANOVA/PRINTE statement within the GLM procedure of SAS. Correlation coefficients were classified as strong (*r* > 0.6), moderate (*r* between 0.4 and 0.6), or weak (*r* < 0.4), respectively.

## Results

### Animal performance, feed intake, and feed efficiency

Summary statistics show animals on test had an average DMI of 10.29 kg/d (SD = 1.46), ADG of 1.37 kg/d (SD = 0.28), G:F of 0.13 kg of BW gain/kg of DMI (SD = 0.02), RFI of 0.00 kg DM/d (SD = 0.77), final live weight of 594.93 kg (SD = 74.25), age of slaughter of 523.56 d (SD = 46.98), and CW of 333.14 kg (SD = 43.99).

Comparisons among RME grouping, sex, and genotype (sire breed maturity), for animal performance, feed intake, and feed efficiency, are displayed in Table 2. In this study, there were no interactions detected (*P* > 0.05) between RME grouping, sex, and genotype for intake, growth, feed efficiency, or carcass composition traits. Indeed, feed intake, growth, bodyweight, feed efficiency measures, and both CW and composition were not different (*P* > 0.05) between the high-, medium-, and low-ranked animals on RME. Steers relative to heifers had a heavier (*P* < 0.05) initial BW, final BW, and CW. Measures of DMI, ADG, FCR, and RFI were not different (*P* > 0.05) among steers and heifers. Animals from EM sires had a greater (*P* < 0.05) ADG than LM. LM sired animals had a heavier CW and MD, but FD and IMF were greater for the EM sired grouping (*P* < 0.05).

**Table 2.** Characterization of feed intake, performance, feed efficiency, ultrasonic measurements and carcass output in finishing beef cattle ranked for residual methane emissions, sex, and genotype

			RME ranking^1^				Sex			Genotype					
Traits^2^	Mean^3^	SD^4^	High, *n* = 84	Medium, *n* = 114	Low, *n* = 84	SEM^5^	Steers, *n* = 128	Heifers, *n* = 154	SEM^5^	Late, *n* = 219	Early, *n* = 63	SEM^5^	*P*-value, RME ranking	*P*-value, sex	*P*-value, genotype
Performance															
Initial weight, kg	475. 7	67.3	472.9	477.4	473.2	8.51	492.3^a^	456. 7^b^	10.3	482.8^a^	466.2^b^	8.0	0.82	0.02	0.04
Metabolic body weight, kg	111.1	10.8	111.2	111.5	110.7	1.43	114.2^a^	108.1^b^	1.8	112.3	110.1	1.4	0.83	0.02	0.09
Final weight, kg	594.9	74.3	599.0	598.8	592.2	11.74	617.2	576.2	15.1	602.4	590.9	11.4	0.70	0.06	0.19
Average daily gain, kg	1.4	0.3	1.4	1.4	1.3	0.05	1.4	1.3	0.1	1.3^a^	1.4^b^	0.1	0.17	0.34	0.04
Feed intake and efficiency															
Dry matter intake, kg/d	10.29	1.46	10.56	10.29	10.26	0.19	10.52	10.22	0.22	10.21	10.53	0.18	0.29	0.36	0.1
G:F, kg	0.13	0.02	0.14	0.13	0.13	0.00	0.14	0.13	0.01	0.13	0.13	0.00	0.21	0.55	0.69
RFI, kg DM/d	0.00	0.77	0.16	0.03	0.10	0.09	0.00	0.20	0.08	-0.08^a^	0.28^b^	0.08	0.48	0.12	<0.01
Ultrasonic measurements															
Fat depth, mm	5.1	1.9	5.5	5.7	5.6	0.4	4.9	6.3	0.5	4.5^a^	6.7^b^	0.4	0.58	0.06	<0.0001
Muscle depth, mm	76.0	7.4	74.5	75.8	75.4	1.6	76.1	74.4	2.2	78.4^a^	72.1^b^	1.6	0.32	0.60	<0.0001
Intramuscular fat, %	6.0	1.4	6.2	6.5	6.3	0.3	6.5	6.2	0.4	5.8^a^	6.9^b^	0.3	0.22	0.64	<0.0001
Carcass characteristics															
Carcass weight, kg	333.1	44.0	328.2	334.7	331.8	7.5	345.6^a^	317.6^b^	9.9	340.5^a^	322.7^b^	7.3	0.46	0.04	<0.001

^1^High, RME was >0.5 SD above the mean; medium, RME was ±0.5 SD above and below the mean; low, RME was >−0.5 SD below the mean.

^2^G:F, gain to feed ratio; RFI, residual feed intake.

^3^Overall trait mean.

^4^Overall trait standard deviation.

^5^SEM, pooled standard error.

^a,b^Least squares means within main effect and a row with different superscripts differ.

### Enteric methane and carbon dioxide output

**Figure 1.**
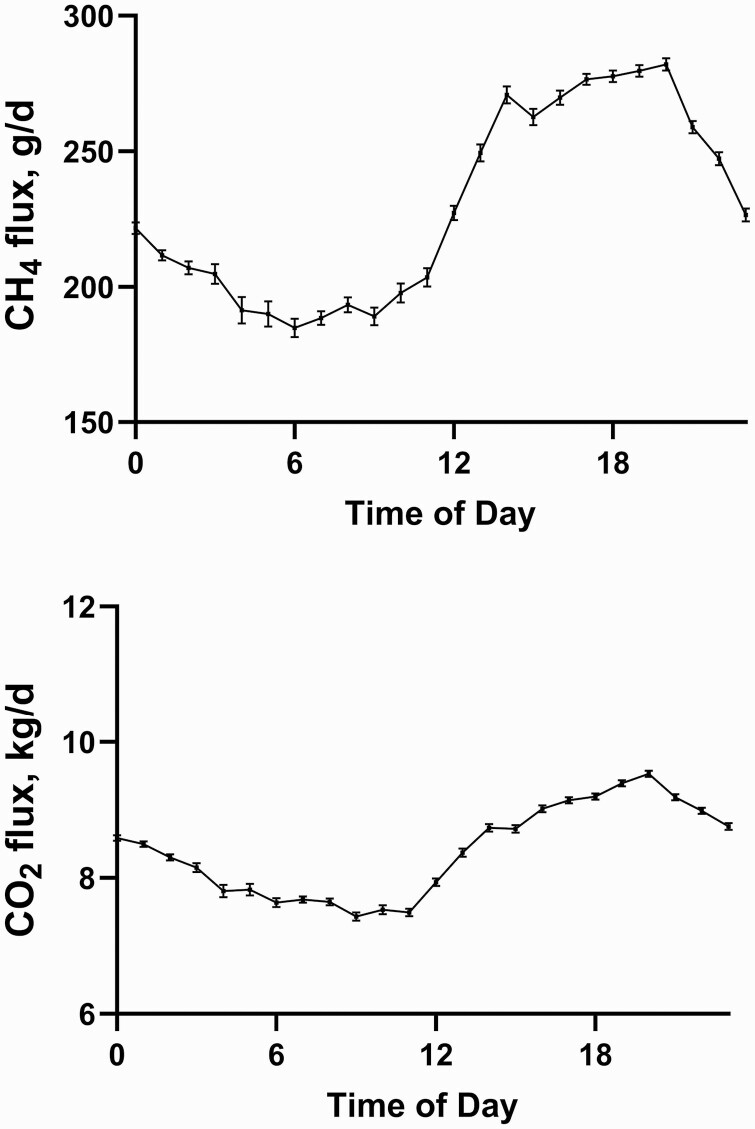
Diurnal pattern of daily methane (CH_4_) and carbon dioxide (CO_2_) emissions. Error bars indicate SEM.

On average, 87.8% of the visits to the GEM were >3 min in length with a mean of 59 valid recordings (i.e., >3 min in length) obtained for each animal. The mean number of valid recordings ranged from 54 to 70 recordings per group of cattle with the highest average valid recordings per animal (70) obtained at a ratio of animals to GEM of 25:1. Animal visitation to the GEM averaged 2.81 times per day (SD = 0.61) during the 21 d enteric emissions measurement period. The average number of daily drops of bait feed was 19.9 drops/d/animal throughout the methane measurement period and ranged from 9.1 to 27 drops/d/animal. During the enteric emissions measurement period, animals had an average daily MDMI of 10.46 kg/d (SD = 1.53), consumed 9.84 kg/d (SD = 1.55) of TMR, and received 0.62 kg/d (SD = 0.13) of concentrate from the GEM. On average, for the high, medium, and low RME groups, the GEM supplemented concentrate account for 5.6%, 6.2%, and 6.3% of total DMI during the emissions measurement period, with no difference observed between the groups (*P* > 0.05). Summary statistics show a mean DME of 229.18 g/d (SD = 45.96), DCE of 8.42 kg/d (SD = 1.02), MY of 22.07 g/kg of DMI (SD = 4.06), MI 0.70 g/kg of CW (SD = 0.15), and MADG 171.67 g/kg of ADG (SD = 40.73). Summary statistics, along with comparisons among RME grouping, sex, and genotype are reported in Table 3. The diurnal pattern of enteric emissions throughout the measurement period is presented in Figure 1.

**Table 3.** Characterization of enteric emissions and methane traits in finishing beef cattle ranked for residual methane emissions, sex, and genotype

			RME ranking^1^				Sex			Genotype					
Traits^2^	Mean^3^	SD^4^	High, *n* = 84	Medium, *n* = 114	Low, *n* = 84	SEM^5^	Steers, *n* = 128	Heifers, *n* = 154	SEM^5^	Late, *n* = 219	Early, *n* = 63	SEM^5^	*P*-value, RME ranking	*P*-value, sex	*P*-value, genotype
DME, g/d	229.2	46.0	265.0^a^	224.0^b^	184.4^c^	8.8	232.0	217.0	12.1	226.4	222.5	8.7	<0.0001	0.38	0.30
DCE, kg/d	8.4	1.0	8.8^a^	8.3^b^	8.1^c^	0.2	8.6	8.2	0.3	8.4	8.4	0.2	<0.0001	0.39	0.92
RME, g/d	0.00	34.1	38.0^a^	-0.1^b^	-40.3^c^	1.8	-0.7	-0.9	1.8	0.6	-2.2	1.6	<0.0001	0.94	0.25
RME_CO2_, g/d	0.00	30.2	24.6^a^	0.7^b^	-31.2^c^	2.4	-1.2	-2.7	2.3	0.6	-4.6	2.1	<0.0001	0.65	0.11
MY, g/kg DMI	22.10	4.1	25.2^a^	21.6^b^	17.7^c^	0.7	21.9	21.1	1.0	21.9^a^	21.1^b^	0.7	<0.0001	0.59	0.01
MADG, g/kg ADG	171.7	40.7	191.3^a^	167.1^b^	144.1^c^	6.6	166.8	168.1	8.5	173.8^a^	161.2^b^	6.4	<0.0001	0.91	0.02
MI, g/kg CW	0.70	0.15	0.81^a^	0.67^b^	0.57^c^	0.03	0.68	0.69	0.05	0.67^a^	0.70^b^	0.03	<0.0001	0.83	0.01

^1^High, RME was >0.5 SD above the mean; medium, RME was±0.5 SD above and below the mean; low, RME was >−0.5 SD below the mean.

^2^DME, daily methane emissions; DCE, daily carbon dioxide emissions; RME, residual methane emissions; RME_CO2_, residual methane emissions calculated with carbon dioxide; MY, methane yield; MADG, methane emissions per kg of ADG; MI, methane intensity.

^3^Overall trait mean.

^4^Overall trait standard deviation.

^5^SEM, pooled standard error.

^a,b,c^Least squares means within main effect and a row with different superscripts differ.

No interactions were detected (*P* > 0.05) between RME grouping, sex, and genotype for any methane or carbon dioxide phenotypes in this study. Low RME animals produced 17.69% and 30.4% less (*P* < 0.05) DME in comparison to animals ranked as medium and high for RME, respectively. Similarly, the low RME group had a lower (*P* < 0.05) DCE than animals ranked as medium and high. Low RME animals had the lowest (*P* < 0.05) MY and MI of the RME groups. A difference of 29.73% and 29.63% for MY and MI was detected among the low and high RME groups, respectively. In addition, the low RME animals produced the least (*P* < 0.05) methane per unit of growth, of the RME groups. No differences among any of the methane phenotypes (*P* > 0.05), including both RME and RME_CO2_, were observed among steers and heifers. No difference in DME, DCE, and RME was detected between genotypes.

### Association analysis among traits associated with methane output and animal productivity

**Figure 2.**
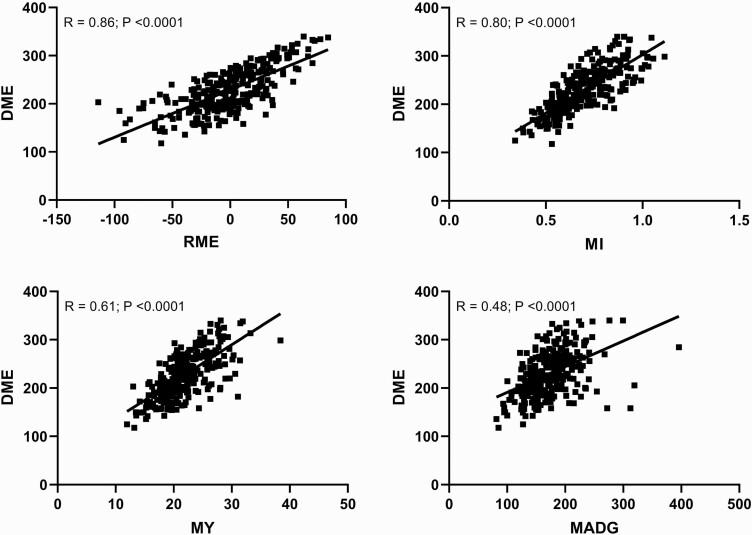
The relationship of daily methane emissions (DME) with residual methane emissions (RME), methane intensity (MI), methane yield (MY), and methane emissions per kg of ADG (MADG).

Correlation coefficients among the methane traits investigated in this study are presented in Table 4. The relationship of DME with RME, MI, MY, and MADG is portrayed in Figure 2. DME were positively correlated (*P* < .0001) with MY, MI, MADG, RME, and RME_CO2_. Among the methane phenotypes, RME was the strongest predictor of daily methane output (*r* = 0.86; *P* < .0001). Between the residual methane traits RME and RME_CO2_ were strongly associated with each other (*r* = 0.86; *P* < .0001), but RME had the stronger correlations with MY (0.89 vs. 0.77) and MI (0.86 vs. 0.78). All three methane ratio traits (MY, MI, and MADG) were positively correlated (*P* < .0001). Positive associations were observed between DCE with DME, RME, and MI.

**Table 4.** Correlations coefficients among methane and carbon dioxide traits

Traits^1^	DME	DCE	RME	RME_CO2_	MY	MI
DME	−					
DCE	0.63***					
RME	0.86***	0.26***				
RME_CO2_	0.76***	−0.02	0.86***			
MY	0.61***	−0.01	0.89***	0.77***		
MI	0.80***	0.23***	0.86***	0.78***	0.73***	
MADG	0.48***	0.08	0.55***	0.57***	0.57***	0.49***

^1^DME, daily methane emissions; DCE, daily carbon dioxide emissions; RME, residual methane emissions; RME_CO2_ , residual emissions production calculated with carbon dioxide; MY, methane yield; MI, methane intensity; MADG, methane emissions per kg of ADG.

***P < 0.001.

**Figure 3.**
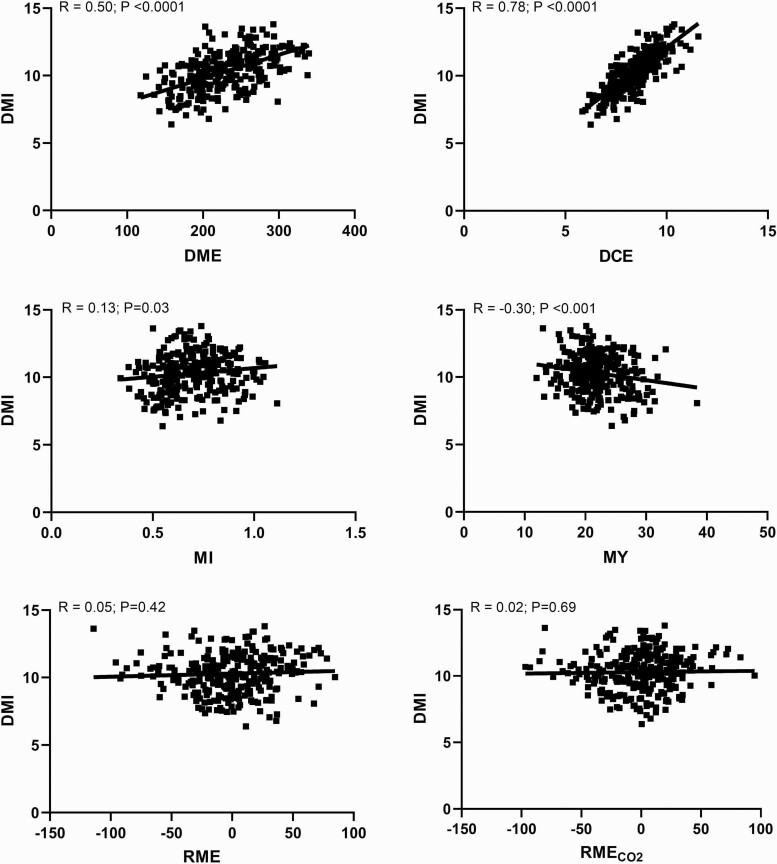
The relationship of dry matter intake (DMI) with daily methane emissions (DME), daily carbon dioxide emissions (DCE), methane intensity (MI), methane yield (MY), residual methane production (RME), and residual methane production with carbon dioxide (RME_CO2_).

Correlation analysis among methane traits with intake, growth, and feed efficiency is presented in Table 5. The relationship of DMI with DME, DCE, MI, MY, RME, and RME_CO2_ is portrayed in Figure 3. The methane traits RME and RME_CO2_ were not associated (*P* > 0.10) with any of the production traits (DMI, ADG, CW, MD, FD, IMF, G:F, or RFI). MY was negatively associated (*P* < 0.05) with DMI, ADG, CW, FD, IMF, G:F, and RFI. MI was positively correlated with DMI, ADG, and RFI and negatively associated with CW and MD (*P* < 0.05). MADG was negatively correlated (*P* < 0.05) with DMI, ADG, and G:F (*P* < 0.05). DCE had a strong positive relationship (*P* < 0.05) with DMI (*r* = 0.78), ADG (*r* = 0.45), and CW (*r* = 0.67).

**Table 5.** Correlations coefficients of intake, performance, feed efficiency traits, and body composition measures with methane traits

Traits^1^	DME	RME	RME_CO2_	MY	MI	MADG
DMI, kg	0.50***	0.05	0.02	−0.30***	0.13*	−0.14*
Average daily gain, kg	0.31***	0.08	0.00	−0.13*	0.13*	−0.63***
Carcass weight, kg	0.31***	−0.03	−0.05	−0.18**	−0.29***	0.00
Muscle depth, mm	0.13*	0.00	−0.01	−0.04	−0.21***	0.08
Fat depth, mm	0.14*	−0.03	0.00	−0.16**	0.00	−0.05
Intramuscular fat, %	0.03	−0.07	−0.07	−0.17**	−0.08	−0.05
G:F	−0.05	0.09	−0.03	0.14*	0.04	−0.66***
RFI	0.23***	−0.01	0.04	−0.24***	0.31**	0.16**

^1^DME, daily methane emissions; DCE, daily carbon dioxide emissions; RME, residual methane emissions; RME_CO2_, residual methane emissions calculated with carbon dioxide; MY, methane yield; MI, methane intensity; MADG, methane emissions per kg of ADG; G:F, gain to feed; RFI, residual feed intake.

*P < 0.05.

**P < 0.01.

***P < 0.001.

### Ruminal fermentation parameters

Comparisons of fermentation parameters among RME grouping, sex, and animal genotype are presented in Table 6. No interactions were detected (*P* > 0.05) between RME grouping, sex or animal genotype for any of the fermentation parameters reported in this study.

**Table 6.** Characterization of rumen fermentation profile in finishing beef cattle ranked for residual methane emissions, sex, and genotype

			RME ranking^1^				Sex			Genotype					
Rumen fermentation^2^	Mean	SD	High, *n* = 84	Medium, *n* = 114	Low, *n* = 84	SEM^3^	Steers, *n* = 128	Heifers, *n* = 154	SEM^3^	Late, *n* = 219	Early, *n* = 63	SEM^3^	P-value, RME ranking	*P*-value, sex	*P*-value, genotype
pH	6.8	0.3	6.8	6.8	6.8	0.1	6.8	6.8	0.1	6.8	6.8	0.1	0.48	0.76	0.10
Total SCFA, mM	124.2	34.4	134.5^a^	120.9^b^	119.9^b^	7.2	119.8	130.4	8.9	123.4	126.8	6.9	0.02	0.39	0.54
Acetate, %	74.3	6.7	73.6	73.1	73.5	1.5	71.9	74.9	1.9	73.7	73.1	1.4	0.85	0.26	0.51
Propionate, %	13.0	4.3	13.0^a^	14.0^a^	14.5^b^	1.2	14.4	13.2	1.5	13.7	13.9	1.1	0.04	0.59	0.66
Butyrate, %	7.8	2.6	8.0	7.8	7.1	0.7	7.7	7.5	0.9	7.5	7.7	0.69	0.10	0.89	0.56
A:P	5.7	1.4	6.7^a^	5.8^b^	5.7^b^	0.6	5.2	7.0	0.8	6.3	5.9	0.6	0.03	0.11	0.31
Hydrogen production, mM	663.7	157.2	688.6^a^	622.9^b^	630.7^b^	37.6	626.5	668.3	48.0	654.8	640.0	36.5	0.03	0.54	0.56

^1^High, RME were >0.5 SD above the mean; medium, RME was ±0.5 SD above and below the mean; low, RME was >−0.5 SD below the mean.

^2^A:P, acetate to propionate ratio.

^3^SEM, pooled standard error.

^a,b^Least squares means within main effect and a row with different superscripts differ.

High RME animals had a greater (*P* < 0.05) total SCFA production in comparison to the medium and low groups. The low RME group had a greater (*P* < 0.05) propionate % in comparison to the high group; however, animals in the high group had a greater (*P* < 0.05) butyrate % compared with both medium and low animals. No difference (*P* > 0.05) in rumen fluid pH, acetate %, A:P ratio or rumen fluid pH was observed among the RME groups. Animals ranked as high had the greatest (*P* < 0.05) theoretical H production of the RME groups. No differences in any of the fermentation associated variables among animal sex or genotype was found (*P* > 0.05).

Correlation analysis of fermentation parameters with all methane traits is reported in Table 7. Total SCFA production had a positive correlation (*P* < 0.05) with DME, RME, RME_CO2_, MY, and MI. Acetate % was positively (*P* < 0.05) correlated with MY and MI. Propionate % was negatively associated (*P* < 0.05) with all methane traits namely DME, RME, RME_CO2_, MY, MI, and MADG. Both RME and RME_CO2_ were positively associated with higher A:P ratio (*P* < 0.05). Butyrate % and theoretical H production were positively correlated (*P* < 0.05) with DME, RME, RME_CO2_, MY, MI, and MADG.

**Table 7.** Correlations coefficients of methane traits with rumen fermentation parameters

Traits^1^	pH	Total SCFA, mM	Acetate, %	Propionate, %	Butyrate, %	A:P	H
DME	0.09	0.19*	−0.08	−0.23**	0.25***	0.07	0.20**
DCE	0.05	−0.01	0.04	0.03	−0.09	−0.10	−0.03
RME	0.08	0.19*	−0.08	−0.25***	0.34***	0.18*	0.22**
RME_CO2_	0.09	0.24**	−0.10	−0.36***	0.41***	0.22**	0.24**
MY	0.08	0.20**	−0.19*	−0.18*	0.37***	0.09	0.23**
MI	0.05	0.28***	−0.18*	−0.18*	0.30***	0.06	0.28***
MADG	0.05	0.12	−0.08	−0.26***	0.24**	0.27***	0.16*

^1^DME, daily methane emissions; DCE, daily carbon dioxide emissions; RME, residual methane emissions; RME_CO2_, residual methane emissions calculated with carbon dioxide; MY, methane yield; MI, methane intensity; MADG, methane emissions per kg of ADG; A:P, acetate to propionate ratio; H, theoretical H production.

*P < 0.05.

**P < 0.01.

***P < 0.001.

## Discussion

Reducing methane emissions from domesticated cattle will be key to achieving a sustainable growth in global food production. Over the past decade, there has been increased international interest in the use of genetic selection as part of a methane mitigation solution for the ruminant livestock sector (Wall et al., 2010; Pickering et al., 2015; de Hass et al., 2017; Beauchemin et al., 2020). However, while the selection of animals solely on DME has the greatest potential to decrease enteric emissions, this is likely to have ramifications for animal productivity, due to the positive relationship between methanogenesis and feed intake (Wall et al., 2010; Pickering et al., 2015; de Hass et al., 2017). Consequently, researchers have proposed alternative indices for ranking the methanogenic potential of an animal. For example, RME has been advocated as having an optimal balance as a trait in identify low emitting animals, while, due to its independence from voluntary feed intake and BW, not impacting on these important drivers of profitability (Herd et al., 2014). However, prior to the completion of the current study, there was a paucity of information available surrounding the implications of ranking beef cattle for RME, on enteric emissions, ruminal fermentation, animal productivity, and carcass output.

Multiple methane phenotypes were evaluated and the values recorded in the present experiment for average DME, along with MY and MI were consistent with previous studies investigating enteric emissions using the GEM technology in beef cattle fed under intensive ad libitum rearing conditions (Arthur et al., 2017; Bird-Gardiner et al., 2017). For example, an average DME of 195.2 and 202.5 g/d was observed by Arthur et al. (2017) and Bird-Gardiner et al. (2017), with the slight increase in emissions observed in this study, likely due to higher proportion of forage in the diet. Additionally, daily animal visitation to the GEM throughout the methane measurement period was within the range (1.3 to 5.08 visits/d) reported by others (Velazco et al., 2016; Alemu et al., 2017; Arthur et al., 2017; Renand et al., 2019) and further strengthens the validity of the methane recording technique implemented in this experiment. The absolute range and differences in growth, performance, feed efficiency, and carcass data between animal sexes and genotypes were comparable to previous production values generated from the same feed efficiency performance test center over the preceding 10 yr (Crowley et al., 2010; Kelly et al., 2011; Kelly et al., 2019; Lahart et al., 2020). DME were positively correlated with feed intake, growth, and carcass output, in line with previous studies (Bird-Gardiner et al., 2017; Renand et al., 2019). There were no differences in DME among the sexes and genotypes, likely explained by the similar level of feed intake and methane bodyweights between the groups, with differences in MI between the breed types due to the increased carcass output observed in LM relative to EM breeds over the finishing period.

Methane ratio traits, such as MY have been the traditional selection approach in identifying high or low emitting animals, as the traits were observed to be independent from any associations with feed intake or BW, when open-circuit respiration chambers and restricted feed intake were implemented as part of the standard operating procedure for quantifying enteric emissions (Herd et al., 2014; Donoghue et al., 2016). However, data generated as part of this study and others (Herd et al., 2016a; Bird-Gardiner et al., 2017; Renand et al., 2019) investigating enteric emissions under various ad libitum feeding regimes, akin to that of a commercial farm setting, indicate the existence of an antagonistic relationship between ratio expressions of methane output and traits of economic importance. For example, the present study observed an unfavorable negative correlation with MY and DMI and equally, all ratio expressions of methane output (MY, MI, and MADG) were correlated with the individual metric of animal performance utilized as a denominator trait in their calculation. Therefore, the applicability of data generated from feed restricted animals to inform methane mitigation breeding strategies is questioned, due to unfavorable associations of ratio expressions of methane output with economically important traits observed under ad libitum feeding conditions.

Alternatively, the selection and ranking of animals on the basis of RME as part of methane mitigation program has been suggested to overcome these limitations associated with ratio-based methane traits and animal productivity, while also maintaining the potential to reduce all indices of methane output (Herd et al., 2014). In support of this, RME were the only methane trait observed to be truly independent of animal production, but positively correlated with enteric emissions in this and other studies where ad libitum feeding was employed (Bird-Gardiner et al., 2017; Renand et al., 2019). In addition, the coefficient of determination for RME in this study is similar to that reported for feedlot steers by Bird-Gardiner et al. (2017). RME were also the best predictor of DME in this experiment and strongly associated with all traditional ratio expressions of methane output. Animals phenotypically ranked as low for RME, in comparison to their high counterparts, produced 30% less DME showing that large interanimal inherent variation exists for this trait. Similarly, low RME animals had a lower MY and MI, producing ~30% less methane per unit of feed intake or CW, in comparison to the high RME group. The reduction in all methane phenotypes in the low RME group occurred in the absence of any adverse effect on animal performance further emphasizing the merit of RME in identifying animals truly divergent for methane output, irrespective of productivity. The acceptance of any methane abatement selection program within the livestock sector will be underpinned by its relationship with on farm profitability (Beauchemin et al., 2020). The phenotypic evidence in this study, supported by genetic correlations and moderate heritability estimates of RME presented by others, albeit under restricted feeding conditions (Donoghue et al., 2016; Manzanilla-Pech et al., 2016), suggests the ruminant livestock sector could reduce the volume of enteric methane emissions in future generations of livestock, without compromising animal productivity, through selection for low RME animals as part of a balanced breeding index or an environmentally focused sub index. Indeed, any mitigation selection program will further benefit from estimations of the heritability and genetic correlations among methane traits under more industry relevant, ad libitum feeding conditions.

Moreover, recently, some authors have advocated for the use of DCE as a proxy for DMI due to the linear relationship observed among both traits (Herd et al., 2016b; Arthur et al., 2018; Donoghue et al., 2020). The strong correlation with DMI, observed here and elsewhere (Arthur et al., 2018), is indicative of the potential benefit of DCE to act as a proxy for feed intake. Indeed, Renand et al. (2019), in forage fed cattle, advocated the potential to calculate RME with CO_2_ in absence of feed intake measures and reported RME_CO2_ to be a good predictor of RME and free from any association with DMI or BW. Concurring, in the present experiment RME_CO2_ maintained similar associations to that of RME with feed intake, growth, feed efficiency, carcass output, and all methane phenotypes. Due to the expense of ongoing accurate determinations of DMI and difficulties in the measurement of the trait at pasture, there may be credence for the use of DCE as a proxy for feed intake when investigating DME and RME. However, the accuracy of DCE as an indicator of feed intake will need to be further evaluated across different dietary regimes and stages of the production cycle.

Ruminal methanogens primarily synthesize methane from H_2_ and CO_2_ with both substrates produced as end products of the microbial fermentation of ingested feed (Moss et al., 2000). Methane is a known byproduct of microbial fermentation with emissions influenced by hydrogen dynamics in the rumen and shifts in microbial fermentation pathways (Janssen, 2010). Indeed, methanogenesis is believed to acquire a homeostatic role in the rumen, by preventing the accumulation of excessive amounts of H_2_ (Morgavi et al., 2010). Ruminal propionate production is considered a competitive hydrogen sink to methanogenesis, with butyrate often considered a net contributor to ruminal hydrogen (Janssen, 2010). In addition, the rumen acetate:propionate ratio is a recognized indicator of an animal’s methanogenic capabilities (Williams et al., 2019). Our data suggest, differences in microbial fermentation pathways particularly the proportion of propionate and butyrate, along with acetate: propionate profile, in the rumen to be among the definitive factors influencing divergence in methane output observed between high- and low-ranked RME animals. Members of both the bacterial and methanogen rumen community are known to influence VFA production and methanogenesis (Kittelmann et al., 2014; Shi et al., 2014; Wallace et al., 2015; Shabat et al., 2016; Auffret et al., 2017; Danielsson et al., 2017; Tapio et al., 2017) making it imperative that further efforts are implemented to identify the key ruminal microbes and methanogenic mechanisms associated with RME to facilitate a greater understanding of the trait. In addition, the increased total SCFA and theoretical H production observed in high RME suggest differences in RME could be influenced by rumen digestibility. Therefore, further studies investigating the relationship of RME with ruminal digestibility and retained energy are warranted.

## Conclusion

RME were the best predictor of DME and were the only methane trait observed to be independent of animal productivity. Ranking cattle in terms of RME, resulted in an ~30% difference between high and low emitting animals for DME, MY, and MI. Differences in methane output among the RME groups were associated with shifts in ruminal hydrogen dynamics resulting from a varied expression of microbial fermentation pathways associated with propionate production. Further in depth rumen microbial analysis is needed to ascertain the key microbes associated with phenotypic and/or genetic divergence for RME in order to facilitate the identification of potential microbial based biomarkers associated with the trait.

## Abbreviations

ADF: acid detergent fiber
ADG: average daily gain
BW: body weight
CG: contemporary group
CH_4_: methane
CO_2_: carbon dioxide
CP: crude protein
CW: carcass weight
DM: dry matter
DMI: dry matter intake
DME: daily methane emissions
EM: early maturing
G:F: gain to feed
GEM: GreenFeed emissions monitoring system
IMF: intramuscular fat
LM: late maturing
ME: metabolizable energy
NDF: neutral detergent fiber
RFI: residual feed intake
SCFA: short chain fatty acids
TMR: total mixed ration
VFA: volatile fatty acid
